# Doege-Potter syndrome associated to metastatic solitary fibrous tumor

**DOI:** 10.4322/acr.2021.412

**Published:** 2022-12-13

**Authors:** Matheus de Oliveira Andrade, Nathália da Cruz de Sousa, Paulo Siqueira do Amaral, Samantha Cabral Severino da Costa, Luiz Guilherme Cernaglia Aureliano de Lima, Delmar Muniz Lourenço, Olavo Feher

**Affiliations:** 1 Universidade de São Paulo (USP), Instituto do Câncer do Estado de São Paulo, Faculdade de Medicina, Departamento de Oncologia Clínica, São Paulo, SP, Brasil; 2 Universidade de São Paulo (USP), Instituto do Câncer do Estado de São Paulo, Faculdade de Medicina, Departamento de Endocrinologia, São Paulo, SP, Brasil; 3 Universidade de São Paulo (USP), Instituto do Câncer do Estado de São Paulo, Faculdade de Medicina, Departamento de Patologia, São Paulo, SP, Brasil

**Keywords:** Solitary Fibrous Tumors, Paraneoplastic Syndromes, Hypoglycemia

## Abstract

Solitary fibrous tumor (SFT) is a rare fibroblastic mesenchymal neoplasm with an estimated annual incidence of 0.35 per 100,000 individuals. Doege-Potter syndrome is a paraneoplastic syndrome related to solitary fibrous tumor clinically characterized by hypoglycemia, occurring in less than 5% of cases. Herein, we report a case of metastatic SFT associated with recurrent severe hypoglycemia. A 43-year-old male with a noncontributory medical history presented with a painless and progressive growing mass in the right thigh. The histological evaluation rendered the diagnosis of SFT, and tumor resection was performed. One year after the operation, on the oncological follow-up, he was admitted to the emergency unit, manifesting an early-morning seizure associated with a severe hypoglycemia. The laboratory findings of non-islet cell tumor hypoglycemia (NICTH) in the background of a relapsed metastatic solitary fibrous tumor were consistent with the diagnosis of Doege-Potter syndrome. Hepatic embolization associated with oral glucocorticoid was an efficient palliative treatment to control the hypoglycemic crisis and allow hospital discharge.

## INTRODUCTION

Solitary fibrous tumor (SFT) is a fibroblastic mesenchymal neoplasm, previously classified as hemangiopericytoma. It is a rare tumor, with an estimated annual incidence of 0.35 per 100,000 individuals.^1^

The pleura is the most involved site, and intrathoracic tumors account for approximately 30% of cases. The main extrapleural site is intraabdominal, including the peritoneum, retroperitoneal soft tissue, and pelvic visceral sites. Less than 10% of SFTs arise from deep tissues of the extremities and abdominal wall.^2^

The diagnosis of SFT is based on histopathological features. The conventional marker in immunohistochemistry is CD34, although it lacks specificity since some other mesenchymal tumors may also express this protein (e.g., gastrointestinal stromal tumors). Other non-specific markers include CD117, DOG1, CD99, desmin and Bcl-2. The most sensitive and specific marker is STAT6, derived from the *NAB-STAT6* fusion gene, which is recognized as a key factor in SFT tumorigenesis. Strong nuclear STAT6 staining reliably differentiates SFT from other tumors, except from dedifferentiated liposarcomas.^3^^,^ 4

An important paraneoplastic syndrome related to solitary fibrous tumors is hypoglycemia (Doege-Potter syndrome), which occurs in less than 5% of cases. Its pathophysiology is related to the secretion of large insulin-like growth factor 2 (IGF-2).^5^

Herein, we report a case of metastatic SFT associated with recurrent severe hypoglycemia.

## CASE REPORT

**Figure 1:**
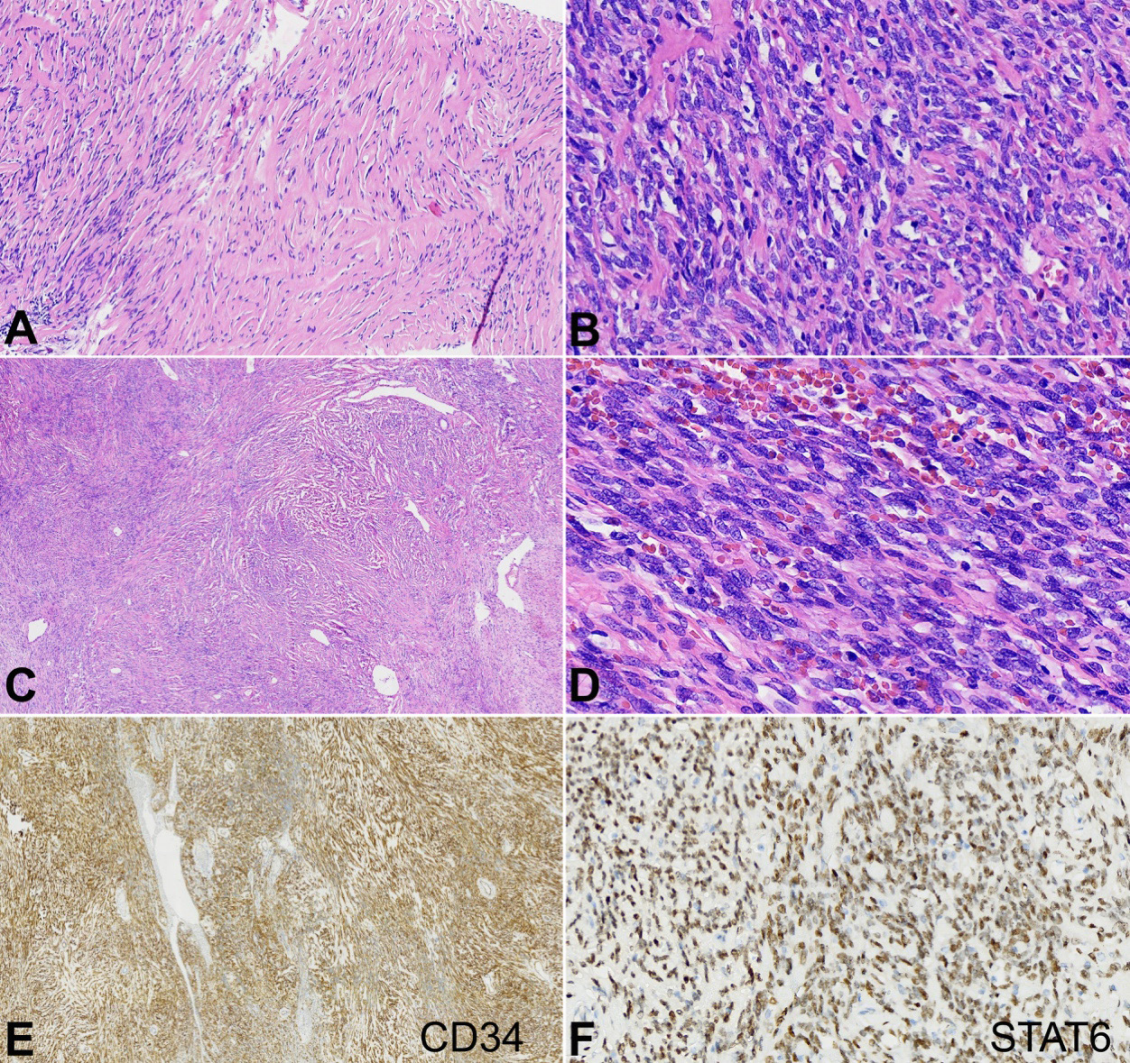
Photomicrographs of the resected tumor of the right thigh revealed a solid fusocellular mesenchymal neoplasm, with: **A –** sclerosing and hyalinizing fibroblastic pattern, exhibiting alternating cellularity (HE, 100x); **B –** including hypercellular nodular areas (HE, 200x); and **C –** pericytic growth, evidenced by tortuous vessels with staghorn pattern (HE, 20x); **D –** Neoplastic cells were relatively monomorphic, exhibited a fibroblastic cytological appearance, with oval nuclei and scarce cytoplasm, low cytological grade and limited hyperchromasia (HE, 400x); Immunohistochemical analysis showed: **E –** positivity for CD34 (40x); and **F –** STAT6, nuclear pattern (200x).

A 43-year-old male with no medical history presented with a painless mass in the right thigh, with progressive growth. The patient sought medical attention, and a biopsy was performed. It revealed a spindle cell neoplasm with high cellularity and atypia that favored aggressive biological behavior. The immunohistochemical evaluation showed CD34, CD99, Bcl-2, and STAT6 positivity and negativity for S-100 and epithelial membrane antigen (EMA), thus confirming the diagnosis of a solitary fibrous tumor**.** As a research protocol, the oncologic treatment was initiated with neoadjuvant radiotherapy, with a total dose of 4000cGy, in 5 fractions. Tumor resection was then performed. Anatomopathological features of the resected tumor of the right thigh are displayed in Figure 1.

One year after the operation, he was admitted to the emergency unit, manifesting an early-morning seizure associated with a glucose level of 33 mg/dL (reference range: 70-99 mg/dL). His initial laboratory findings were unremarkable: HbA1c, renal, hepatic, thyroid, and adrenal function tests were normal. After 6-hour of fasting, the patient’s serum glucose level was 13 mg/dL, IGF1 23 ng/mL, insulin < 0.6 µU/mL, and C-peptide 0.05 nmol/L. Low serum insulin and IGF1 levels suggested high serum IGF2 levels (Table 1).

**Table 1:** Laboratory workup

**Test**	**Values**	**Reference range**
Glucose* (mg/dL)	13	70 - 99 mg/dL
Insulin*(μU/mL)	< 0.6	2.6 – 24.9 μU/mL
C-peptide* (ng/mL)	0.05	1.1 – 4.4 ng/mL
IGF-1* (ng/mL)	23	74.9 – 216.4 ng/mL
HbA1c (%)	5.0	4.1 – 6.0%
TSH (μIU/mL)	2.2	0.27 – 4.20 μIU/mL
Serum cortisol (μg/dL)	25.7	5.0 – 25 ug/dL
AST (U/L)	19	<37 U/L
ALT (U/L)	18	<41 U/L
INR	1.13	0.95 – 1.2
Albumin (g/dL)	3.4	3.4 – 3.9 g/dL
Bilirubin (mg/dL)	0.61	0.2 – 1.0 mg/dL
Creatinine (mg/dL)	0.39	0.7 – 1.2 mg/dL

* Venipuncture during hypoglycemia (6-hour fasting period).

IGF: insulin-like growth factor; TSH: thyroid-stimulating hormone; AST: aspartate aminotransferase; ALT: alanine transaminase; INR: international normalized ratio; HbA1c: glycated hemoglobin.

**Figure 2:**
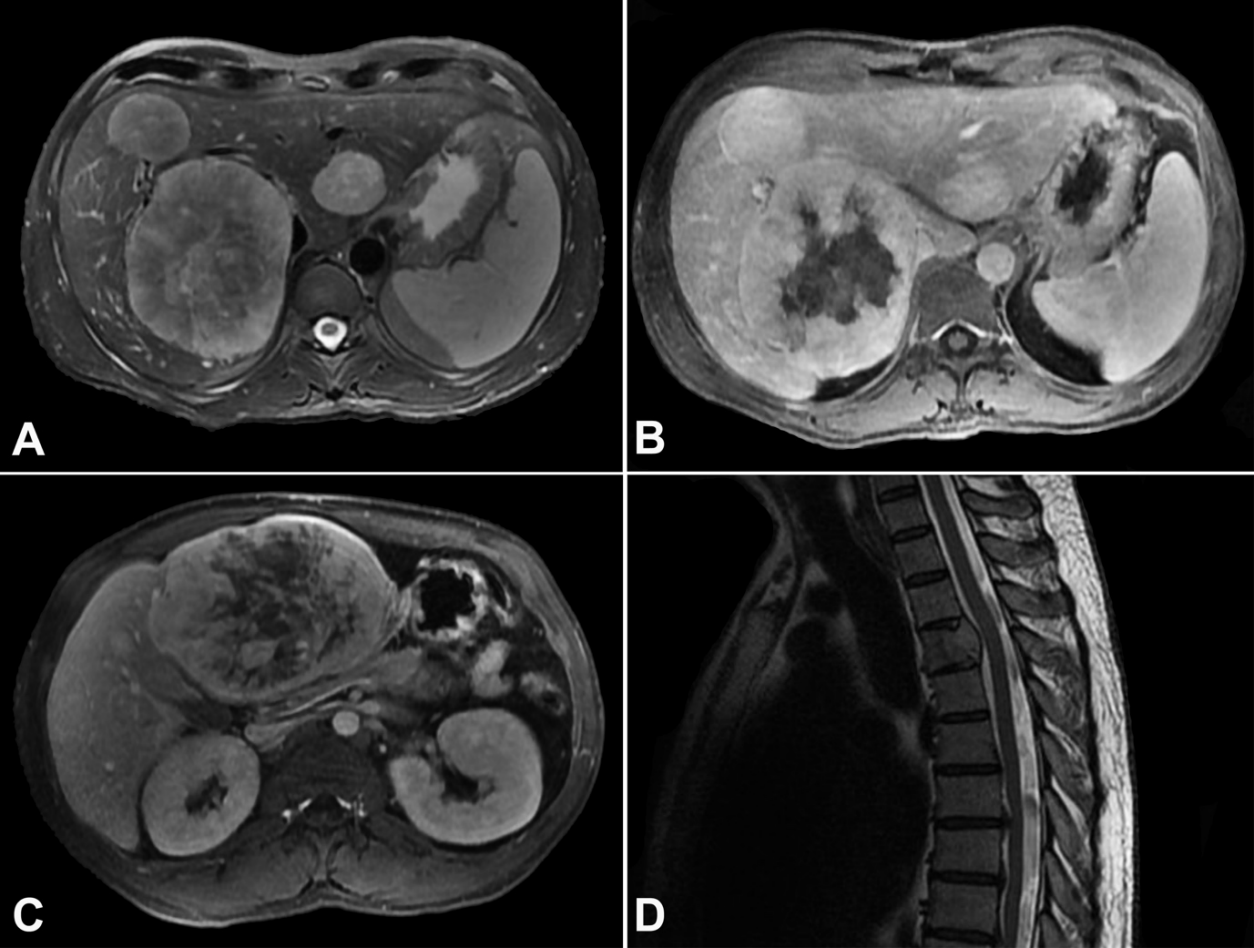
MRI scans in axial planes show multiple hepatic nodules distributed over both lobes. **A –** (T2-weighted sequence), the largest hypervascularized with extensive central necrosis, measuring 11.2 x 8.8 cm in segment VII/VII; **B –** (DCE sequence) and in segment III, measuring 12.5 x 9.3 cm; **C –** (DCE portal sequence); **D –** osteolytic lesions in the vertebral body of D4 (T2-weighted sequence in the sagittal plane), with a soft tissue component determining compression on the dural sac.

**Figure 3:**
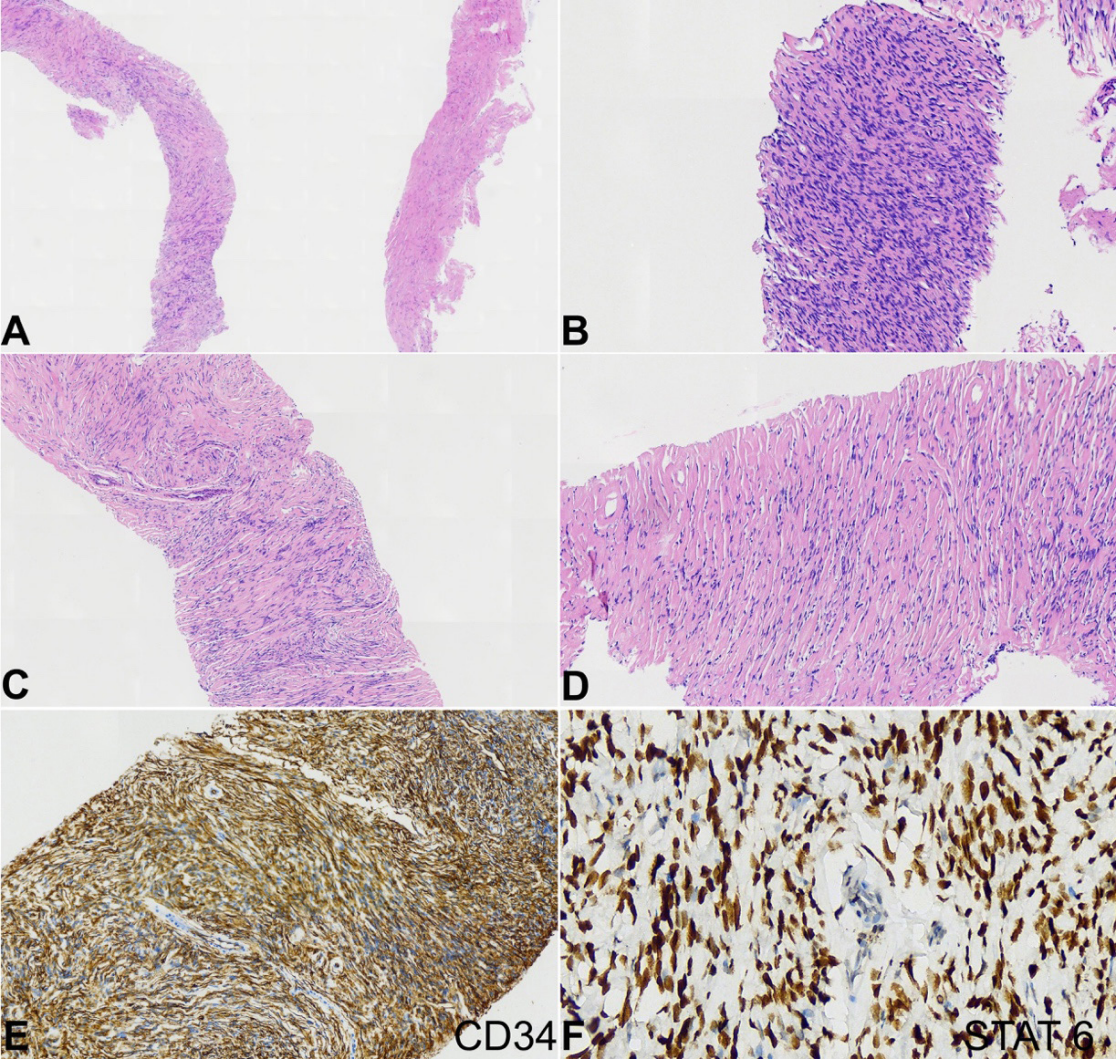
Photomicrographs of the percutaneous liver biopsy showed: **A –** monomorphic and fibrosclerosing proliferative mesenchymal lesion with alternating cellularity (HE, 20x); **B –** including hypercellular areas (HE, 100x); **C –** with poorly defined cellular arrangement (HE, 40x); **D –** some ectatic vessels with hyalinized wall (HE, 100x). Immunohistochemical examination showed: **E –** consistent positivity for CD34 (100x); **F –** STAT6, nuclear pattern (400x).

Imaging studies documented more than ten liver nodules, the largest in segments III (12.5 x 9.3 cm) and VII/VIII (11.2 x 8.8 cm), and osteolytic lesions in the vertebral bodies of D4 and L3, suggestive of metastases (Figure 2). Percutaneous liver biopsy confirmed hepatic metastases of SFT (Figure 3).

The laboratory findings of non-islet cell tumor hypoglycemia (NICTH) in the background of a metastatic solitary fibrous tumor, were consistent with the diagnosis of Doege-Potter syndrome.

Despite adequate oral intake, the patient required continuous intravenous infusion of 10% dextrose in high doses to maintain normoglycemia. Treatment with corticosteroids (prednisone 40 mg/day) was started with a reduction of constant intravenous infusion of 10% dextrose. He underwent transarterial hepatic embolization, aiming at treating dominant metastatic lesions, which allowed complete discontinuation of intravenous glucose infusion and hospital discharge. The patient performed a second elective transarterial hepatic embolization and has been followed for six months without recurrence of hypoglycemia.

## DISCUSSION

Surgical resection with negative margins is the mainstay of therapy for the localized solitary fibrous tumor. The behavior of SFT is usually indolent, and local or distant recurrence is not a common feature. However, it is estimated that 10 to 30% of SFTs may recur. Long-term follow-up of these patients is important since late recurrence up to 20 years after the initial presentation is reported.^6^^-^ 8

The management of advanced and metastatic SFT is challenging, and few studies on this rare clinical entity exist. The response to chemotherapy is usually poor and reserved for unresectable tumors and metastatic disease. Traditional chemotherapy agents for soft tissue sarcoma, such as ifosfamide, anthracyclines, and dacarbazine, are considered options for metastatic disease, although they usually have low response rates. Antiangiogenic agents, such as tyrosine kinase inhibitors (i.e., pazopanib, sorafenib), may exhibit antitumor efficacy and constitute options for progressive disease.^9^^-^ 11

The key molecular feature of SFTs is the fusion of two genes, *NAB2* (NGFI-A binding protein 2) and *STAT6* (signal transducer and activator of transcription 6). The product of the *NAB2-STAT6* fusion gene is a chimeric transcription factor that constitutively activates *NAB2* target genes. *IGF2* is considered one of the target genes, with its expression dysregulated by the chimeric transcription factor. This explains the frequency of paraneoplastic hypoglycemia associated with solitary fibrous tumors (Doege-Potter syndrome).^3^

Doege-Potter syndrome is a differential diagnosis of non-islet-cell tumor hypoglycemia (NICTH). NICTH is a rare paraneoplastic syndrome often discovered incidentally in the pursuit of the cause of hypoglycemia. Most come from mesenchymal tumors with low malignant potential. It involves anomalous production of IGF-2, which will bind to insulin receptors, causing non-ketotic hypoinsulinemic hypoglycemia. The typical laboratory findings of NICTH include glycemia < 55 mg/dL, insulin < 3 µU/mL, proinsulin < 5 pmol/L, C-peptide < 0.2 nmol/L, beta-hydroxybutyrate < 2.7 mmol/L and the IGF2/IGF1 ratio > 10. Low IGF‑1 levels in patients with NICTH are related to chronic attenuation of GH secretion due to the negative feedback of IGF‑2.^8^^,^ 12 ^,^ 13

There is no clear standard of care for managing non-islet-cell tumor hypoglycemia. Ideal treatment includes complete tumor resection. Glucocorticoids are considered the initial medical therapy of choice in cases where the underlying malignancy is not resectable. Their action is based on impairing insulin action, enhancing gluconeogenesis, and increasing clearance of IGF-2. In addition, it is postulated that IGF-2 may act as an autocrine growth factor for the tumor; thus, IGF-2 clearance could impair tumor growth.^14^^,^ 15

There are case reports of Doege-Potter syndrome that have illustrated the resolution of refractory hypoglycemia with glucocorticoid use in inoperable IGF-2-producing SFT.^15^ Furthermore, selective arterial embolization of the tumor was previously reported in other cases, as a therapeutic alternative in unresectable tumors or a palliative measure for paraneoplastic hypoglycemia.^16^^-^ 18 In the present case report, both strategies were combined to ensure adequate glycemic control. Resolution of hypoglycemia was achieved without chemotherapy or other systemic treatments, which usually have low response rates.

Besides imaging tests, follow-up monitoring of glucose metabolism and hypoglycemic symptoms may be useful for detecting tumor recurrence and metastasis.^19^ In our case, the liver and vertebral metastasis diagnosis occurred due to hospital admission with hypoglycemia.

## CONCLUSION

This case is unique in its presentation as a rare paraneoplastic syndrome caused by hepatic and bone metastases from the solitary fibrous tumor. It also highlights the difficulty of management when definitive surgical treatment is not feasible, considering the inefficiency of the available systemic therapy. Hepatic embolization associated with oral glucocorticoid was an efficient palliative treatment to resolve the hypoglycemic crisis and allow hospital discharge.
